# Independent and sensitive gait parameters for objective evaluation in knee and hip osteoarthritis using wearable sensors

**DOI:** 10.1186/s12891-021-04074-2

**Published:** 2021-03-03

**Authors:** Ramon J. Boekesteijn, José M. H. Smolders, Vincent J. J. F. Busch, Alexander C. H. Geurts, Katrijn Smulders

**Affiliations:** 1 Department of Research, Sint Maartenskliniek, Hengstdal 3, 6574 NA Ubbergen, Nijmegen, The Netherlands; 2 Department of Rehabilitation, Donders Institute for Brain, Cognition and Behaviour, Radboud University Medical Center, Nijmegen, The Netherlands; 3 Department of Orthopedic Surgery, Sint Maartenskliniek, Nijmegen, The Netherlands

**Keywords:** Knee osteoarthritis, Hip osteoarthritis, Inertial measurement units, Gait analysis, Dual-task

## Abstract

**Background:**

Although it is well-established that osteoarthritis (OA) impairs daily-life gait, objective gait assessments are not part of routine clinical evaluation. Wearable inertial sensors provide an easily accessible and fast way to routinely evaluate gait quality in clinical settings. However, during these assessments, more complex and meaningful aspects of daily-life gait, including turning, dual-task performance, and upper body motion, are often overlooked. The aim of this study was therefore to investigate turning, dual-task performance, and upper body motion in individuals with knee or hip OA in addition to more commonly assessed spatiotemporal gait parameters using wearable sensors.

**Methods:**

Gait was compared between individuals with unilateral knee (*n* = 25) or hip OA (*n* = 26) scheduled for joint replacement, and healthy controls (*n* = 27). For 2 min, participants walked back and forth along a 6-m trajectory making 180° turns, with and without a secondary cognitive task. Gait parameters were collected using 4 inertial measurement units on the feet and trunk. To test if dual-task gait, turning, and upper body motion had added value above spatiotemporal parameters, a factor analysis was conducted. Effect sizes were computed as standardized mean difference between OA groups and healthy controls to identify parameters from these gait domains that were sensitive to knee or hip OA.

**Results:**

Four independent domains of gait were obtained: speed-spatial, speed-temporal, dual-task cost, and upper body motion. Turning parameters constituted a gait domain together with cadence. From the domains that were obtained, stride length (speed-spatial) and cadence (speed-temporal) had the strongest effect sizes for both knee and hip OA. Upper body motion (lumbar sagittal range of motion), showed a strong effect size when comparing hip OA with healthy controls. Parameters reflecting dual-task cost were not sensitive to knee or hip OA.

**Conclusions:**

Besides more commonly reported spatiotemporal parameters**,** only upper body motion provided non-redundant and sensitive parameters representing gait adaptations in individuals with hip OA. Turning parameters were sensitive to knee and hip OA, but were not independent from speed-related gait parameters. Dual-task parameters had limited additional value for evaluating gait in knee and hip OA, although dual-task cost constituted a separate gait domain. Future steps should include testing responsiveness of these gait domains to interventions aiming to improve mobility.

**Supplementary Information:**

The online version contains supplementary material available at 10.1186/s12891-021-04074-2.

## Background

It is well-recognized that osteoarthritis (OA) of the knee or hip impairs gait [1–4]. Indeed, individuals with knee or hip OA walk less during daily life and their quality of gait is compromised [5]. Yet, objective gait assessments are not part of routine clinical evaluation, and gait difficulties in OA are insufficiently captured by patient-reported outcome measures [6–8]. In part, this may be due to limited time available during clinical visits, considering that gait analysis is traditionally conducted in a gait laboratory, making it time consuming and not easily accessible. Recent advances in inertial sensor technology have opened up new avenues to quickly and objectively assess gait quality in a clinical setting.

Small inertial measurement units (IMUs) can be used to quickly and accurately obtain gait parameters without being restricted to a fixed (laboratory) environment [9, 10]. Moreover, compared to gait analysis in a lab, substantially more strides can be collected in a shorter period of time. On the downside, an important issue of gait assessment with IMUs is that it typically results in a large number of outcome parameters, with numerous correlated parameters. For example, many gait parameters share covariance with gait speed [11–15]. Hence, for clinical implementation, it is important to identify gait parameters from independent gait domains that best describe the gait adaptations in individuals with knee and hip OA compared to healthy controls.

So far, ambulatory gait assessments in individuals with knee and hip OA have mostly been limited to simple, straight-ahead walking paradigms [16]. Parameters reflecting more complex and relevant aspects of gait, including dual-task gait, turning, and compensatory trunk motion are less frequently reported in studies using IMUs. Turning and dual-task performance have been shown to be important aspects of daily life ambulation in elderly populations and can easily be assessed using wearable sensors [17–20]. Turning is a common cause of falling in community dwelling elderly, and may be more sensitive to sensorimotor impairments than straight-ahead gait [19, 21]. Dual-task performance, on the other hand, reflects the amount of attentional resources allocated to gait [22]. In order to compensate for gait difficulties caused by OA, a strategy could be to allocate more attention to gait. The extent to which a secondary cognitive task affects gait performance (i.e. dual-task cost (DTC)) may therefore be larger in individuals with OA. A recent scoping review indicated that DTC was not different between individuals with knee OA and healthy controls during quiet standing and forward induced falls [23]. However, DTC during gait has not yet been compared between those groups. A third gap in literature regarding wearable sensors and OA is the lack of attention for upper body movement. Upper body motion is important for maintaining stability, but may also be indicative of compensatory gait changes that reflect OA-related pain or disability [24–26].

The aim of this study was therefore to investigate turning, dual-task performance, and upper body motion in addition to spatiotemporal gait parameters in individuals with knee or hip OA, taking shared covariance between gait parameters into account. More specifically, we aim to test if 1) turning, dual-task gait, and upper body motion constitute independent domains of gait in our sample, and 2) gait parameters in these gait domains can discriminate individuals with knee or hip OA from healthy controls. Together, these findings may contribute to a better understanding of the multidimensional aspects of gait, and how this is affected in knee and hip OA.

## Methods

### Participants

In this cross-sectional, comparative study 78 participants were included. The total study population comprised three groups: individuals with unilateral knee OA (*n* = 25), unilateral hip OA (*n* = 26), and healthy controls (*n* = 27). Samples were derived from a longitudinal study investigating gait before and after total knee and hip arthroplasty that was powered for the comparison of spatiotemporal gait characteristics between individuals 1 year after total knee or hip arthroplasty and healthy controls. Individuals with OA were recruited at the Sint Maartenskliniek and were included if they had both radiological and symptomatic OA and were listed for joint replacement surgery. Participants had to be able to walk for more than 2 min without the use of any assistive device. Exclusion criteria were: 1) expectancy of joint replacement within a year, or symptomatic OA, in another weight-bearing joint than the joint scheduled for surgery, 2) BMI > 40 kg/m^2^, and 3) any other musculoskeletal or neurological impairment interfering with gait or balance. Healthy controls were recruited from the community and did not have a clinical diagnosis of knee or hip OA, nor did they have any pain in the lower extremities. Healthy controls were recruited in the same age range as individuals with OA. Exclusion criteria for healthy controls were the same as for individuals with knee and hip OA. Informed consent was obtained from all participants prior to testing. Ethical approval was obtained from the CMO Arnhem/Nijmegen (2018–4452). All study methods were carried out in accordance with the Declaration of Helsinki.

### Demographic and clinical assessment

Evidence for radiological OA was provided by the Kellgren and Lawrence (KL) score as assessed by experienced orthopedic surgeons [27]. Anthropometric characteristics were obtained during the pre-operative screening visit and were summarized as mass, length, and BMI. For individuals with knee and hip OA, self-reported functioning was assessed using the Knee Injury and Osteoarthritis Outcomes Score (KOOS) or Hip Disability Osteoarthritis Outcome Score (HOOS) [28, 29]. All items were scored on a zero to four Likert scale. For the five subscales, total scores were transformed to a 0–100 scale, with 100 representing best function.

### Gait assessment

**Fig. 1:**
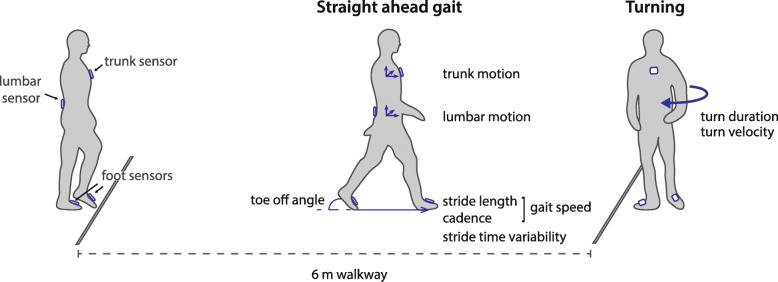
Overview of the experimental set-up. Four IMUs were attached to the dorsum of both feet, lumbar level (L4/L5) of the waist, and the sternum. For 2 min, subjects walked back and forth over the 6 m trajectory, making 180 degree turns

Gait parameters were collected on the same day as the pre-operative screening visit, which took place approximately 1 to 2 months prior to surgery. Four IMUs (*Opal V2, APDM Inc., Portland, OR)* were used to obtain segment accelerations and angular velocities (sample frequency = 128 Hz). Sensors were attached via elastic straps to the dorsum of both feet, the waist (sacrolumbar level), and the sternum (Fig. 1) according to the standardized sensor placement of MobilityLab. Participants walked wearing flat shoes at a self-selected comfortable speed. For a duration of 2 min, participants walked back and forth along a 6-m trajectory making 180° turns (Fig. 1). Two 2-min trials were collected, with and without a secondary cognitive task. The cognitive task consisted of an alternating alphabet task, citing every other letter of the alphabet. Single-task walking was always performed before the dual-task condition. Responses to the cognitive task were recorded by the assessor. Accuracy on the cognitive task was summarized as correct responses (percentage of total responses). DTC was computed as the percentual change of dual-task performance relative to the single-task for the following parameters: gait speed, cadence, stride length, stride time variability, and turn duration.

### Data analysis

Gait parameters were extracted from the raw IMU signals using the commercially available and validated Mobility Lab v2.0 software package [30]. Mobility Lab uses a state space model with causal Kalman filter along with zero velocity updates for optimal orientation estimation. Range of motion metrics were described for both the lumbar and trunk sensors using the gyroscope signals. As such, these measures are representative of the rotation of the sensors, which is caused by the movement of the underlying segments. For parameters where side was relevant (i.e. foot elevation at midswing, lateral step variability, circumduction, foot strike angle, toe off angle, and stance duration), we analyzed the affected leg in individuals with knee or hip OA, whereas for healthy controls the average value from the left and right leg was taken. Gait parameters were initially selected based on reliability, theoretical considerations, and completeness (< 20% missing values). Based on the reliability criterium, we excluded stance and swing duration as percentage of gait cycle [31]. With regard to theoretical considerations, the following decisions were made: 1) in case gait parameters reflected the same outcome (e.g. gait cycle duration and cadence) only one parameter was kept for further analysis, 2) asymmetry parameters were restricted to meaningful parameters (i.e. stride length cannot be asymmetric when walking over a straight path) [32]. DTC of gait parameters that are ratios (i.e. asymmetry values) were not included in order to prevent inflated values, except for stride time variability, due to the substantial number of other studies evaluating this parameter in the context of fall risk [33]. This resulted in twenty-five gait parameters entered into factor analysis to identify correlated outcomes.

Exploratory factor analysis was used to identify independent gait domains explaining the variance in gait performance. Adequacy of the dataset for factor analysis was tested using Barlett’s test of sphericity and the Kaiser-Meyer-Olkin (KMO) test. In case individual KMO values were lower than 0.5, variables were removed from the analysis [34]. The number of factors to be retained for further analysis was determined using the Kaiser criterium (eigenvalue > 1.0) [35]. Subsequently, factor analysis with varimax rotation was performed to obtain orthogonal factor scores. Within a factor, gait parameters were considered relevant when they met a minimum factor loading of 0.5.

For each relevant gait parameter in the obtained factor, effect sizes were computed as standardized mean differences (SMD) for the comparison between the OA groups and healthy controls (knee OA vs healthy controls and hip OA vs healthy controls). The gait parameter with the highest factor loading in combination with an effect size larger than 0.5 was considered non-redundant and sensitive to either knee or hip OA. For these gait parameters, individual datapoints and means with 95% confidence intervals (CI) were constructed using estimation graphs to assess between-group differences [36].

For demographic and clinical parameters, main group effects (3 levels: knee OA, hip OA, healthy controls) were tested using a one-way ANOVA or non-parametric equivalent when assumptions for parametric testing were not met. In case of a significant main effect, a post-hoc comparison was conducted using independent samples Student’s t-test or the non-parametric equivalent. Data was considered statistically significant at an alpha level of 0.05, which was adjusted for multiple comparisons (*n* = 9) for the gait parameters. This resulted in a Bonferroni adjusted alpha level of 0.0056. Data analysis was performed using STATA and custom-written Python scripts incorporating the DABEST library [37].

## Results

### Participant characteristics

Age, sex, and height did not differ between OA groups and healthy controls (Table 1). Individuals with knee OA had – on average – a 9 kg (95% CI: 2–16; *p* = 0.014) higher mass compared to healthy controls. This difference was 12 kg (95% CI: 3–20; *p* = 0.007) between individuals with hip OA and healthy controls. For individuals with knee OA, this translated into a 2.8 kg/m^2^ (95% CI: 0.9–4.7; *p* = 0.005) higher mean BMI compared to the control group, whereas the mean BMI was 2.4 kg/m^2^ (95% CI: 0.1–4.7; *p* = 0.043) higher in individuals with hip OA. Severity of radiographic OA was moderate to severe OA (KL = 3 or 4) in both groups. Furthermore, accuracy on the secondary cognitive task was comparable between individuals with knee (mean: 84%) or hip OA (mean: 87%) and healthy controls (mean: 89%). KOOS and HOOS scores indicated presence of pain, disability, and limited quality of life in individuals with knee and hip OA (Table 1). Gait parameters were based on 32 valid strides (95% CI: 29–36) in individuals with knee OA, 34 valid strides (95% CI: 31–37) in individuals with hip OA, and 30 valid strides (95% CI: 27–34) in healthy participants.

**Table 1:** Demographic and clinical characteristics of all three subject groups

Parameter	Controls (***n*** = 27)	Knee OA (***n*** = 25)	Hip OA (***n*** = 26)	ANOVA main group effect	Post-hoc comparisons
Age (y)	66 [63–68]	64 [61–67]	64 [61–66]	F(2,75) = 0.67, *p* = 0.514	–
Sex (M:F)	13:14	12:13	17:9	χ^2^ (2, *N* = 78) = 2.09, *p* = 0.352	–
Height (m)	1.72 [1.68–1.75]	1.72 [1.68–1.77]	1.76 [1.73–1.80]	F(2,75) = 1.72, *p* = 0.185	–
Mass (kg)	76 [72–80]	84 [79–90]	88 [80–95]	F(2,75) = 4.51, ***p*** **= 0.014**	Knee OA vs HC: mean diff = **9 [2–16**], *p =* 0.014 Hip OA vs HC: mean diff **= 12 [3–20],** *p* = 0.007
BMI (kg/m^2^)	25.7 [24.6–26.8]	28.5 [26.9–30.1]	28.1 [26.0–30.1]	F(2,75) = 3.52, ***p*** **= 0.035**	Knee OA vs HC: mean diff **= 2.8 [0.9–4.7]**, *p* = 0.005 Hip OA vs HC: mean diff **= 2.4 [0.1–4.7]**, *p* = 0.043
KL score (I:II:II:IV)	–	0:0:8:17	0:0:7:19	–	–
DT scores (% correct)	89 [86–92]	84 [79–89]	87 [84–91]	F(2,75) = 1.56, *p* = 0.217	–
**Self-reported outcomes**		**KOOS**	**HOOS**		
1) Symptoms	–	50.9 [42.5–59.3]	41.4 [33.6–49.2]	–	–
2) Pain	–	41.7 [33.8–49.5]	39.6 [34.4–44.8]	–	–
3) Activities of daily life	–	52.9 [44.9–60.9]	39.7 [33.7–45.6]	–	–
4) Sport/ Recreation	–	15.6 [7.9–23.3]	15.1 [10.5–19.8]	–	–
5) Quality of life	–	26.0 [20.4–31.6]	23.6 [17.8–29.3]	–	–

Data are presented as mean [95% CI]. Significant differences are bold

*OA* osteoarthritis, *KL* Kellgren and Lawrence, *BMI* body mass index, *DT* dual-task, *HC* healthy controls, *HOOS* hip disability and osteoarthritis outcome score, *KOOS* knee injury and osteoarthritis outcome score

### Exploratory factor analysis

**Fig. 2:**
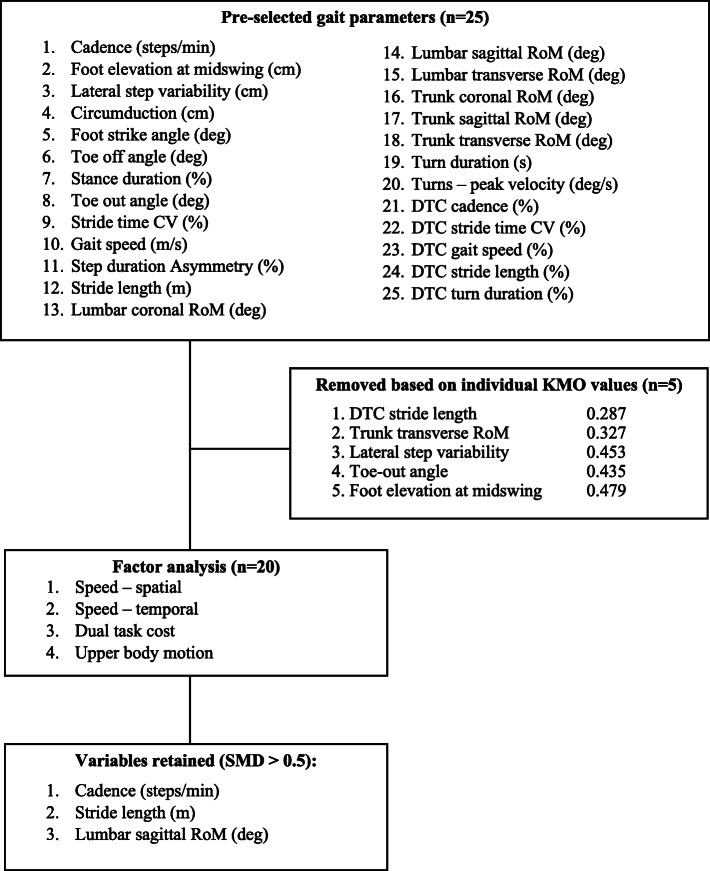
Flowchart describing the selection process of gait parameters. *Note:* foot elevation at midswing = height of the foot sensor at mid-swing, lateral step variability = spatial deviation in the lateral direction of each foot compared to previous steps, circumduction = amount that the foot travels perpendicular to forward movement during the swing phase

Twenty-five gait parameters were entered into the factor analysis (Fig. 2). Based on individual KMO values, the following variables were removed from further analysis: DTC of stride length, trunk transverse range of motion (RoM), lateral step variability, toe-out angle, and foot elevation at midswing. Factor analysis of the remaining twenty parameters yielded four orthogonal factors accounting for 87.8% of the total variance in gait performance (Table 2). The factors were described as speed-spatial, speed-temporal, dual-task cost, and upper body motion. Gait speed had a cross-loading on the factors speed-spatial (0.759) and speed-temporal (0.579). Turning parameters loaded on the factor speed-temporal. In the upper body motion domain, factor loadings of the parameters were relatively low, ranging between 0.53 and 0.61.

**Table 2:** Item loadings obtained from the factor analysis (*n* = 4) with varimax rotation

Gait parameters	Speed-spatial	Speed-temporal	Dual-task cost	Upper body motion
Stride Length (m)	**0.907**	0.270	0.050	−0.000
Gait Speed (m/s)	**0.759**	**0.579**	0.170	−0.062
Foot Strike Angle (deg)	**0.742**	0.120	−0.161	0.257
Toe Off Angle (deg)	**0.628**	0.267	0.129	−0.233
Stride Time CV (%)	−**0.596**	−0.260	− 0.077	−0.051
Cadence (steps/min)	0.203	**0.830**	0.284	−0.163
Turns – Peak velocity (deg/s)	0.420	**0.745**	−0.090	0.102
Turn Duration (s)	−0.453	**−0.704**	0.108	0.092
DTC Cadence (%)	0.067	0.010	**0.935**	0.047
DTC Gait Speed (%)	0.060	0.107	**0.921**	0.057
Lumbar Sagittal RoM (deg)	0.113	−0.159	0.028	**0.611**
Lumbar Transverse RoM (deg)	0.029	0.134	0.131	**0.562**
Trunk Sagittal RoM (deg)	0.008	−0.221	0.111	**0.543**
Trunk Coronal RoM (deg)	−0.049	−0.123	− 0.008	**0.528**
**Explained variance (%)**	**30.0**	**22.5**	**20.7**	**14.6**

Barlett’s test of sphericity confirmed absence of an identity matrix (χ^2^ (190) = 1447.09, *p* < 0.001). Suitability of the dataset was indicated by the Kaiser-Meyer-Olkin measure, which was 0.666. Together the four factors explained 87.8% of the variance in our sample

*CV* coefficient of variation, *DTC* dual-task cost, *RoM* range of motion

### Selection of gait parameters based on effect size

**Fig. 3:**
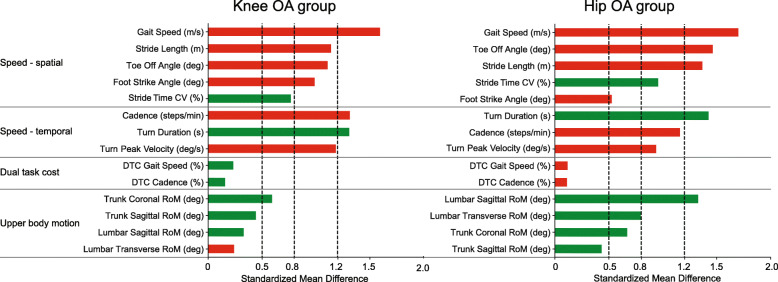
Effect sizes expressed as standardized mean differences of all gait parameters in the different factors for the comparison of healthy controls with individuals with knee OA (left) and individuals with hip OA (right). Red colors indicate OA < healthy controls, green colors represent OA > healthy controls. Please note that gait speed had a cross loading and was also part of the speed-temporal domain. *Note:* CV = coefficient of variation, DTC = dual-task cost, RoM = range of motion

SMDs for the comparison between OA groups and healthy participants are visualized for all gait parameters in Fig. 3. Based on the criterium for effect size, the following gait parameters were selected to represent the corresponding factors: stride length (speed-spatial), cadence (speed-temporal), and lumbar sagittal RoM (upper body motion). Although the factor DTC explained 20.7% of the total variance in gait performance, none of the gait parameters within this factor showed an effect size larger than 0.5 (Fig. 3). Gait speed showed the largest effect size for both the comparison between knee OA and controls (SMD = 1.59) and hip OA and controls (SMD = 1.70). However, due to cross-loadings on factors speed-spatial and speed-temporal, gait speed was not prioritized over stride length and cadence. In addition, many of the gait parameters from the factor speed-spatial and speed-temporal showed large effect sizes (SMD > 0.8) for both group comparisons.

### Between group comparisons of non-redundant gait parameters

**Fig. 4:**
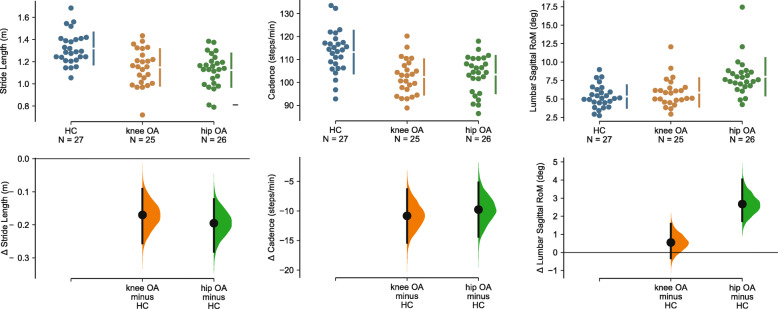
Estimation plots of the mean group differences for stride length, cadence, and lumbar sagittal RoM. In the top panel, dots represent the individual datapoints and bars the mean (± SD). In the bottom panel, the distribution of the mean difference (± 95% CI) for the comparison with healthy controls is visualized. In cases where zero is not in the 95% CI of the mean difference, as indicated by the black bars in the lower panels, data was statistically different at *p* < 0.05

Between-group differences of the selected gait parameters were visualized using estimation plots (Fig. 4). Both individuals with knee and hip OA walked with a lower cadence and with shorter steps. More specifically, compared to healthy controls stride length was 0.17 m (95% CI: 0.09–0.26, *p* < 0.001) lower in individuals with knee OA and 0.20 m (95% CI: 0.12–0.28, *p* < 0.001) lower in hip OA. In addition, cadence was 10.8 steps/min (95% CI: 6.3–15.4, *p* < 0.001) lower in individuals with knee OA and 9.8 steps/min (95% CI: 5.2–14.4, *p* < 0.001) lower in individuals with hip OA. Lumbar RoM in the sagittal plane was 2.7 degrees (95% CI: 1.7–4.4, *p* < 0.001) higher for individuals with hip OA compared to controls, whereas no differences were found between knee OA individuals and healthy controls (mean difference = 0.5 degrees, 95% CI: − 0.33-1.59, *p* = 0.260).

## Discussion

The aim of the present study was to investigate turning, dual-task performance, and upper body motion in addition to spatiotemporal gait parameters in individuals with knee or hip OA. To avoid redundancy of gait parameters, we conducted a factor analysis. Four independent gait domains were identified: speed-spatial, speed-temporal, dual-task cost, and upper body motion. Turning did not constitute its own domain but was related to speed-temporal. Three domains held parameters sensitive to knee or hip OA: speed-spatial (stride length), speed-temporal (cadence), and upper body motion (lumbar sagittal RoM). Dual-task cost was not sensitive to knee or hip OA.

Factor analysis effectively reduced the dimensionality of our dataset from twenty-five gait parameters to four independent domains of gait, including domains related to dual-task gait and compensatory trunk motion. Turning, however, was part of a factor together with cadence. The factors explaining most of the variance in our sample, i.e. speed-spatial and speed-temporal, were both dependent on gait speed (Table 2). In the literature, these factors reflecting the spatial and temporal aspects of gait speed are consistently reported [38–42]. Other factors related to gait are variability [38, 39, 41, 42], asymmetry [39, 41, 42], postural control [39], and trunk motion [40]. Dual-task cost has not previously been evaluated in a factor analysis approach, but may contain unique information about gait that is informative of disease-specific compensations related to the re-allocation of attentional resources. Importantly, dual-task cost and upper body motion are interesting domains as they were independent of gait speed, evidenced by the absence of a cross-loading of gait speed on these domains in our study. Dual-task cost and upper body motion may therefore provide promising gait parameters for clinical evaluation of gait, in addition to the more commonly used speed-related measures.

In our analysis steps, turning parameters were excluded in favor of the stronger factor loading that was obtained for cadence. However, effect sizes for turning were large when comparing both knee and hip OA with healthy participants (SMD > 0.9, Fig. 3). In addition, factor loadings were not substantially lower compared to cadence. Taken together, we are unsure whether this factor represents a combination of gait and turning, or better reflects turning itself. Future research should therefore indicate as to what extent turning parameters are driven by cadence or gait speed, and how meaningful the unexplained variance is for evaluation of physical functioning in individuals with knee and hip OA.

To facilitate assessment of the between-group differences, we opted to select single gait parameters from the independent factor, to represent the respective factor. From the factors that we obtained, only dual-tasking parameters did not discriminate between knee or hip OA and healthy controls (SMD < 0.5). This indicates that, compared to healthy controls, individuals with OA did not need more attentional resources for the motor task. Thus, although gait was affected in OA, this was not compensated by more attentional resources.

Many of the gait parameters that showed large between-group effect sizes (Fig. 3) were grouped either under the speed-spatial or under the speed-temporal domain. This suggests that the two main components determining gait speed, stride length and cadence, are inherently linked with various gait adaptations prominent in individuals with knee and hip OA. As such, gait speed may also be considered as the final common pathway for various gait adaptations, and could be used as a very general, but highly sensitive marker for functional status in individuals with OA. Next to this, our findings further stress the need to take gait speed differences into account when evaluating gait in individuals with OA. More specifically, for parameters that are correlated with gait speed, it may be more appropriate to assess them at a standardized, matched speed, as it may be difficult to separate effects of gait speed from the effects of OA itself [43]. Finally, these findings underline the importance of data reduction techniques when investigating gait using IMUs or motion capture systems, as statistical testing of all gait parameters would increase the probability of finding false positives.

That speed-related gait parameters have good discriminatory capacity in OA has been reported before. Two systematic reviews reported lower gait speed and stride length in individuals with knee and hip OA compared to healthy participants [1, 3]. In studies employing IMUs, similar changes in stride length and cadence were found [25, 44]. In absolute numbers, slight differences with our values can be discerned. Reasons for this may include the relatively short walking distance (6 m) in this study that was necessary to reliably assess turning, versus the longer distances (~ 20 m) that are commonly used. Nevertheless, our findings corroborated previous findings about the discriminatory capacity of stride length and cadence.

In addition to spatiotemporal differences, individuals with hip OA walked with distinct upper body motion, which was most evident in the sagittal plane at the lumbar level. However, upper body motion is difficult to capture by just one parameter, as is illustrated by the relatively low factor loadings lying close together in this domain (Table 1). Altered trunk motion may point toward the use of compensatory strategies to unload the arthritic joint [45]. More specifically, increased pelvic RoM in the sagittal plane may enable more effective anteflexion of the lower limbs and may thereby, to a certain extent, preserve stride length [46]. In addition, anterior pelvic tilt combined with lateral trunk lean can reduce the lever arm between the hip joint center and center of mass [25]. We observed more lumbar sagittal RoM and more RoM of the trunk in the coronal plane in individuals with hip OA compared to healthy controls, in line with previous reports [25, 46]. Unfortunately, the exact reason for the use of these compensatory mechanisms remains speculative and may relate to pain, muscle weakness, or joint instability [47]. Future research should therefore investigate the importance of upper body motion in individuals with OA, to inform us about potential mechanisms underlying these gait adaptations.

With regard to the use of wearable sensors in clinical practice, our study showed that quick and easy gait assessments with wearable sensors are useful for evaluating gait impairments in individuals with knee and hip OA. In comparison to optical motion capture systems, wearable sensors are more feasible for large-scale use and could be utilized to routinely assess physical functioning. From all gait parameters, gait speed was found to be a very general but highly sensitive marker for mobility limitations, combining both the effects on stride length and cadence. Besides the basic spatiotemporal measures, trunk motion and turning appeared to be relevant for individuals with knee and hip OA. We therefore recommend to use sensor configurations that allow to look beyond these basic spatiotemporal parameters. In the future, wearable sensors should also be utilized to their fullest potential to enable remote monitoring at home, which would allow to more accurately capture the habitual gait patterns.

This study had several limitations that merit attention. First, we did not obtain factors representing gait asymmetry or variability, which may have been related to the low number of gait parameters related to those domains that were initially entered into factor analysis. We were therefore limited in our conclusions regarding the potential value of those measures for individuals with knee or hip OA. Second, five potentially valuable gait parameters were removed from further analysis due to sampling inadequacy (KMO value < 0.5). Larger sample sizes are therefore required to identify the potential value of these parameters. Related to this, we did not include demographic or clinical variables in the factor analysis, as this could have affected the accuracy of factor analysis due to the relatively small sample size. Finally, including individuals with isolated, unilateral knee or hip OA was important for our study purposes, although the majority of the OA population have complaints in more than one joint [48]. We expect that widening the inclusion criteria would have resulted in larger differences of OA groups compared to healthy controls, but in less specificity for each OA group. In addition, it is important to note that individuals in this study had end-stage OA and were scheduled for joint replacement. Our results may thus not be representative of gait in individuals with less severe OA.

## Conclusion

In addition to commonly assessed spatiotemporal parameters, this study provided two other relevant domains of gait: dual-task cost and upper body motion. Although dual-task cost provided unique information about gait, our results did not suggest that individuals with knee or hip OA needed more attention for walking than healthy participants. Adaptations in upper body motion were more subtle than stride length and cadence, but may carry important information about compensatory strategies that are most distinctive for individuals with hip OA. Future steps should include evaluation of the responsiveness of these gait parameters to effects of interventions aiming to improve mobility, such as joint replacement surgery. Furthermore, longitudinal monitoring of individuals with knee and hip OA starting at earlier stages of the disease may inform us about the development of these gait adaptations and associated compensations over time.

## Supplementary Information

**Additional file 1.**

## Data Availability

The dataset supporting the conclusions of this article is included within the article and its additional files.

## Abbreviations

OA: Osteoarthritis
IMU: Inertial measurement unit
DTC: Dual-task cost
KL: Kellgren and Lawrence
KOOS: Knee injury and osteoarthritis outcome score
HOOS: Hip disability and osteoarthritis outcome score
KMO: Kaiser-Meyer-Olkin
SMD: Standardized mean difference
CI: Confidence interval
RoM: Range of motion
